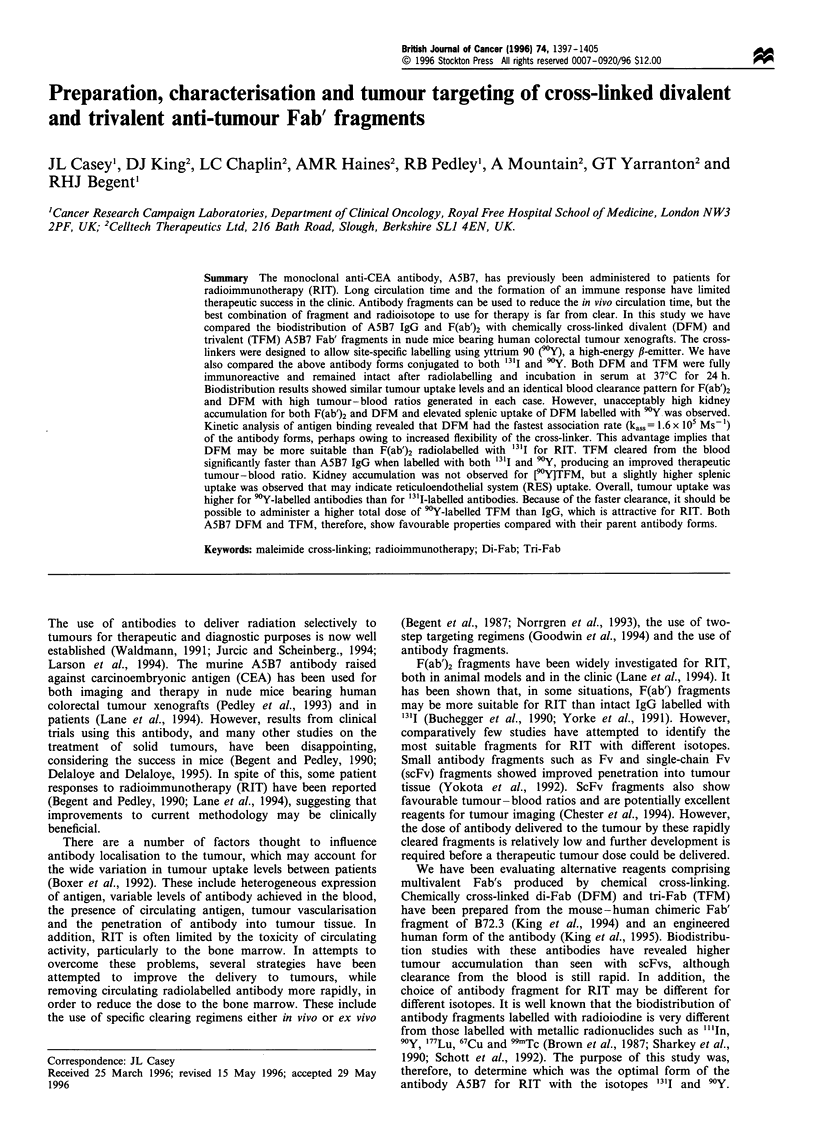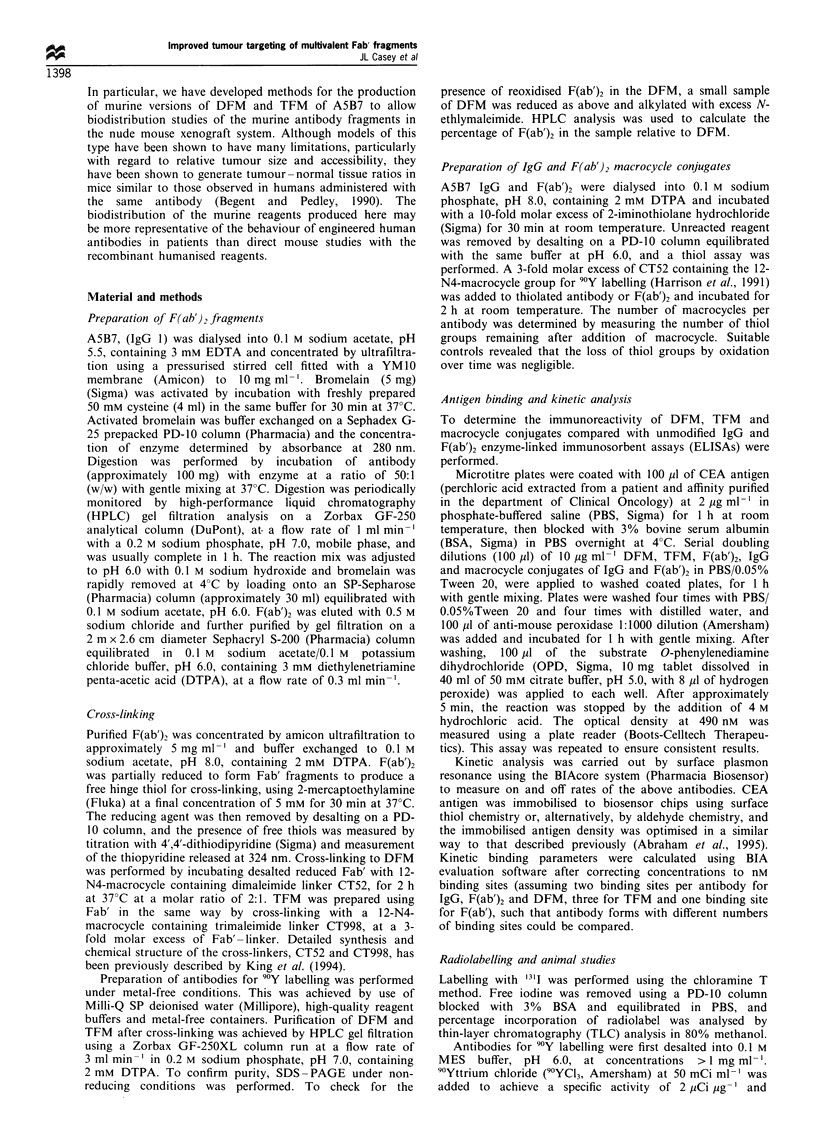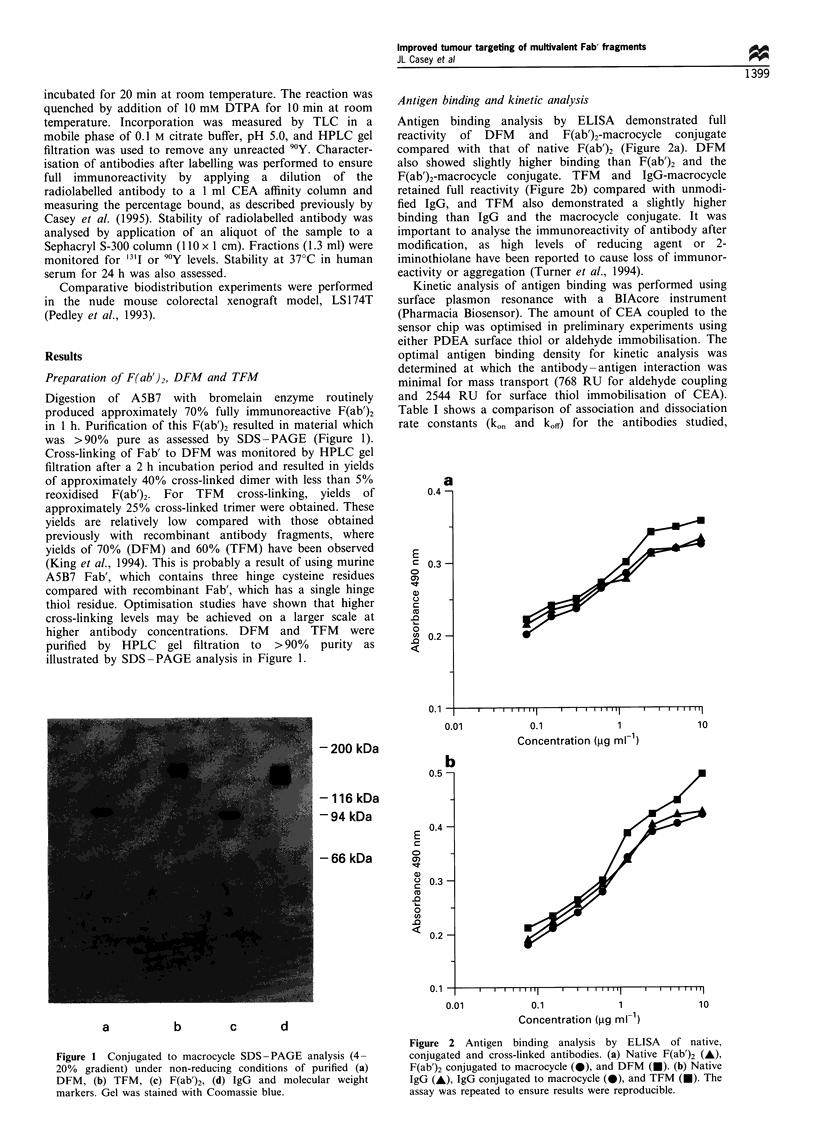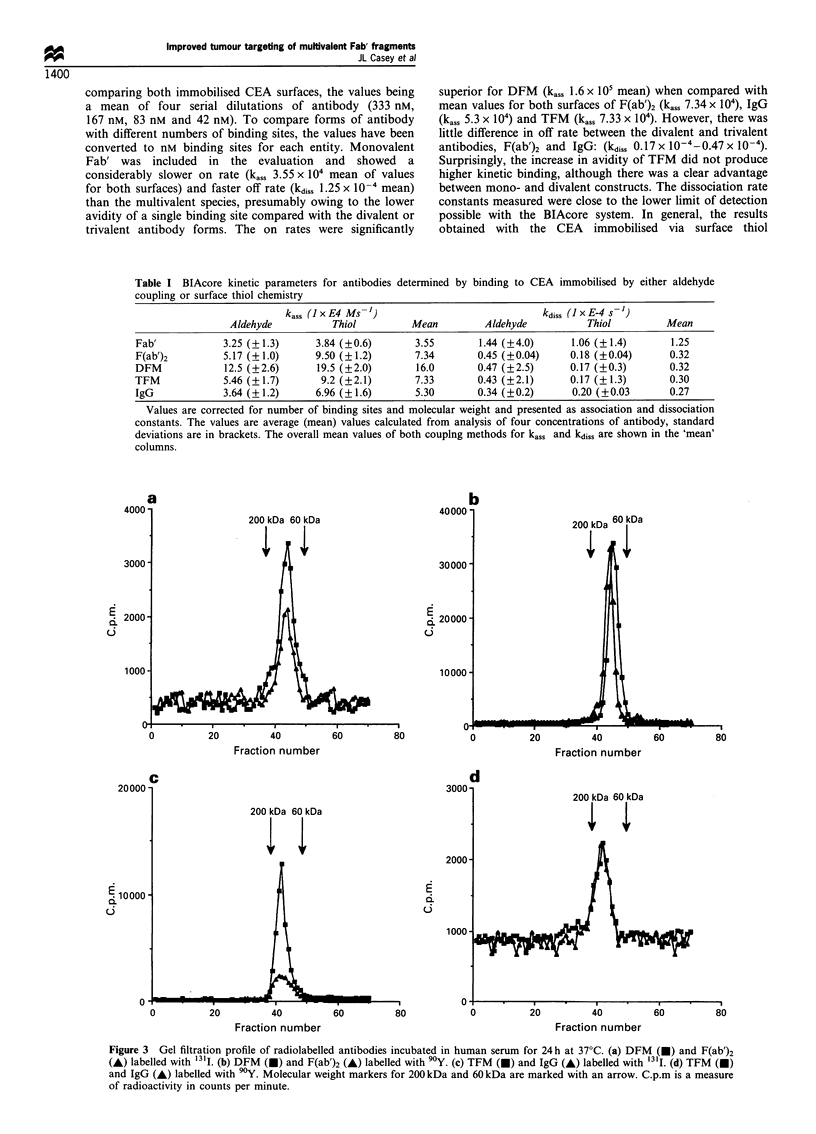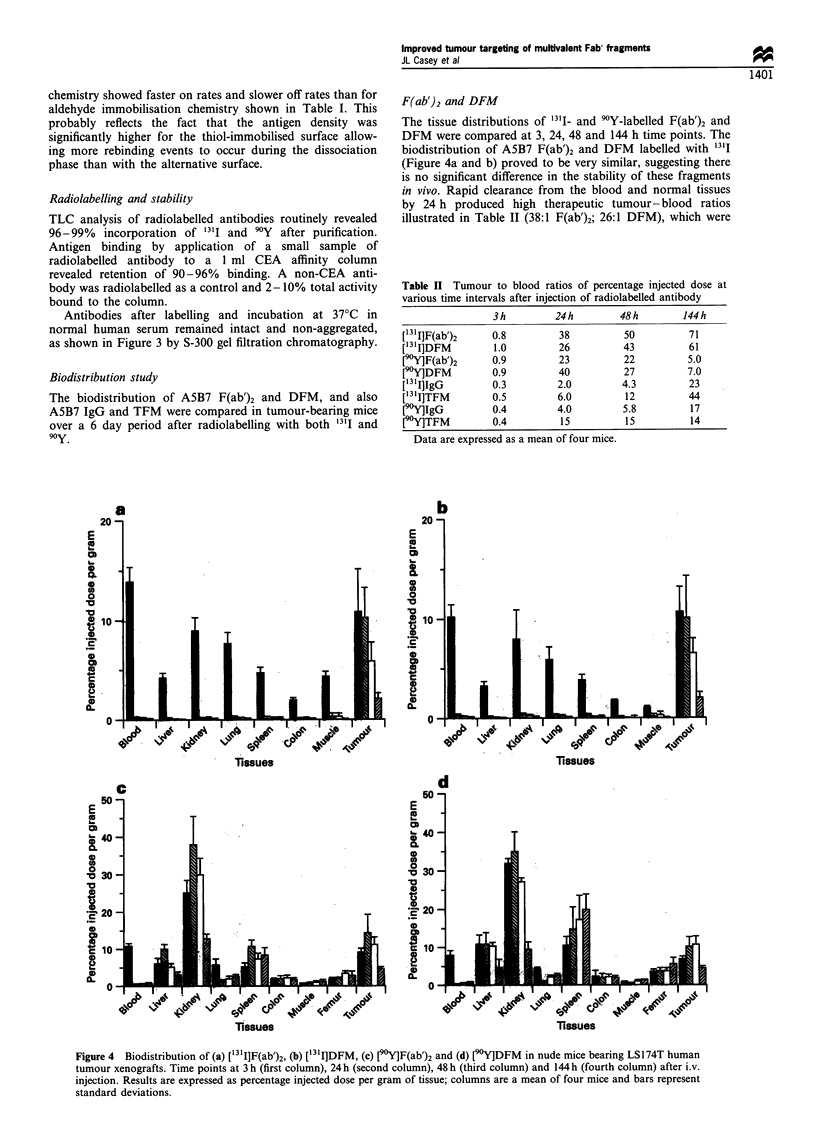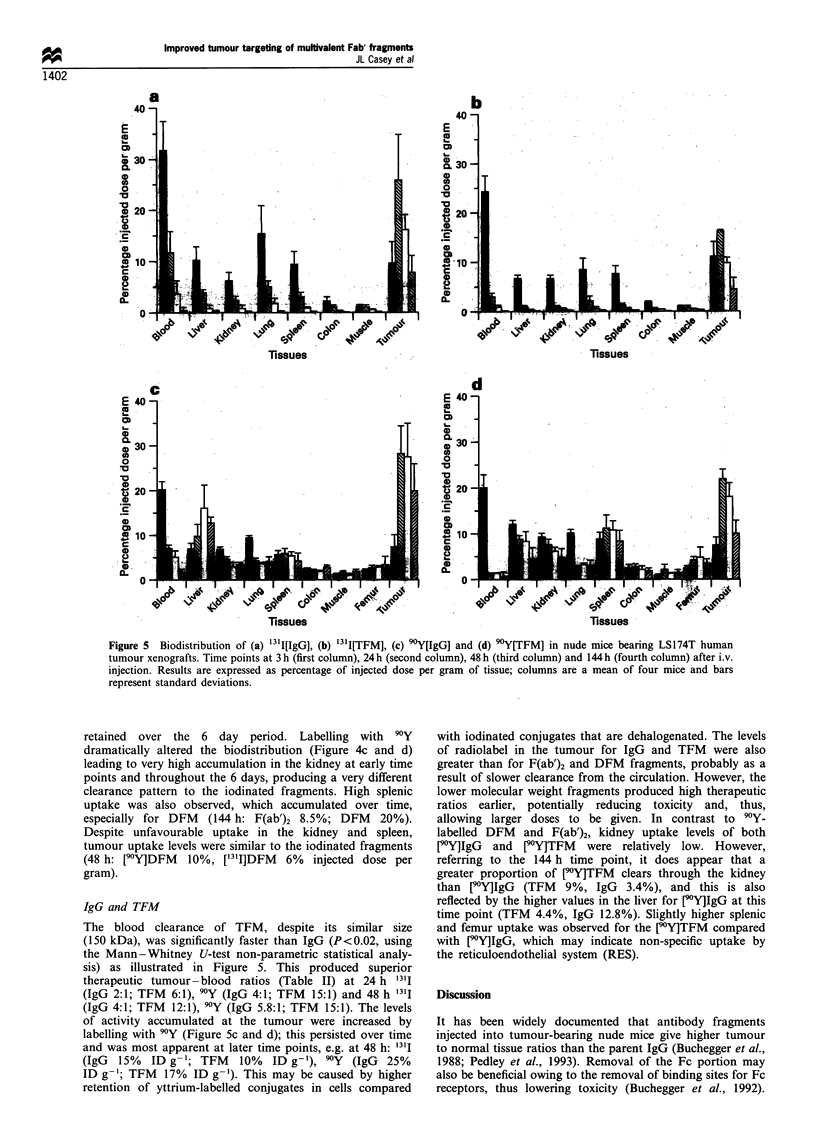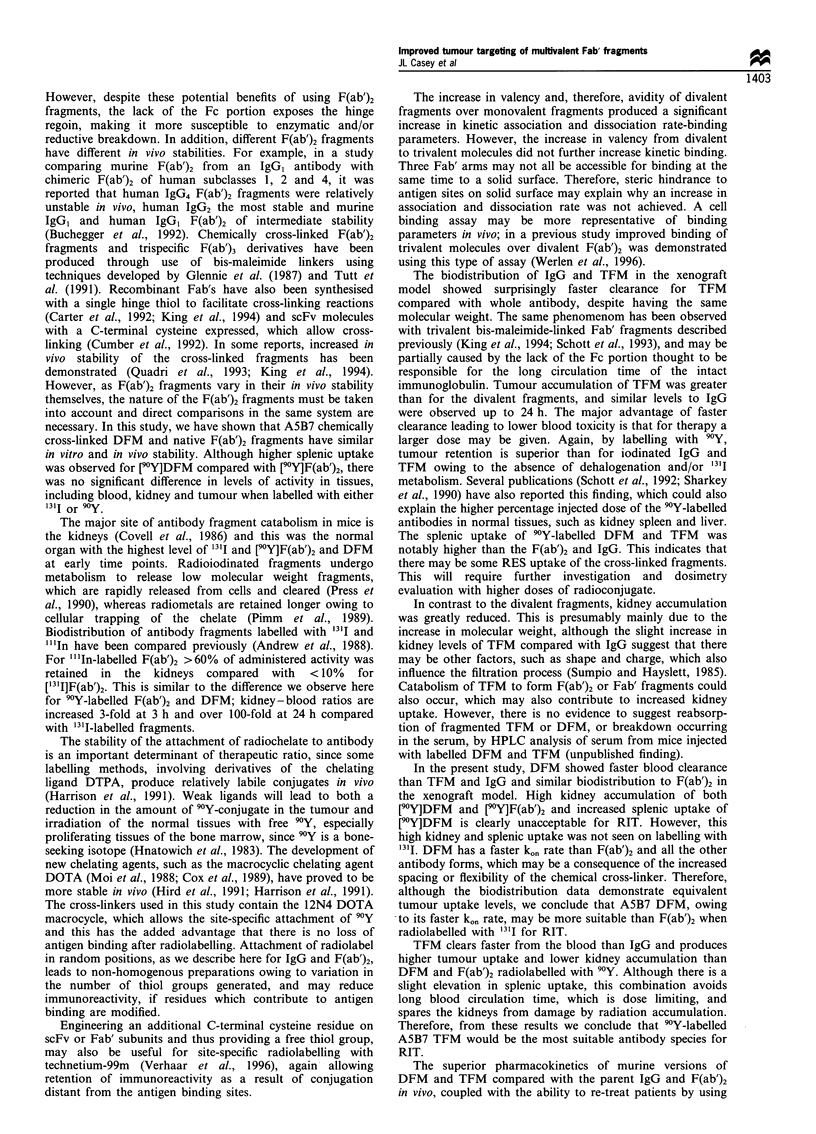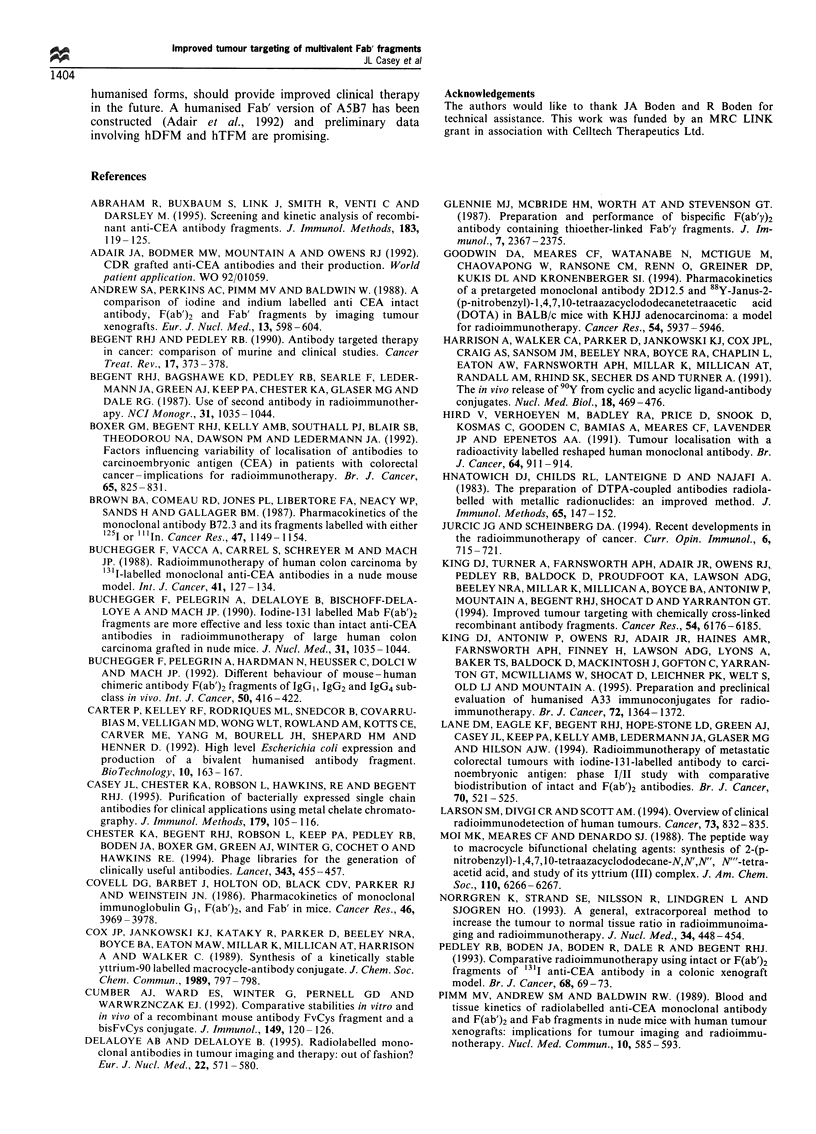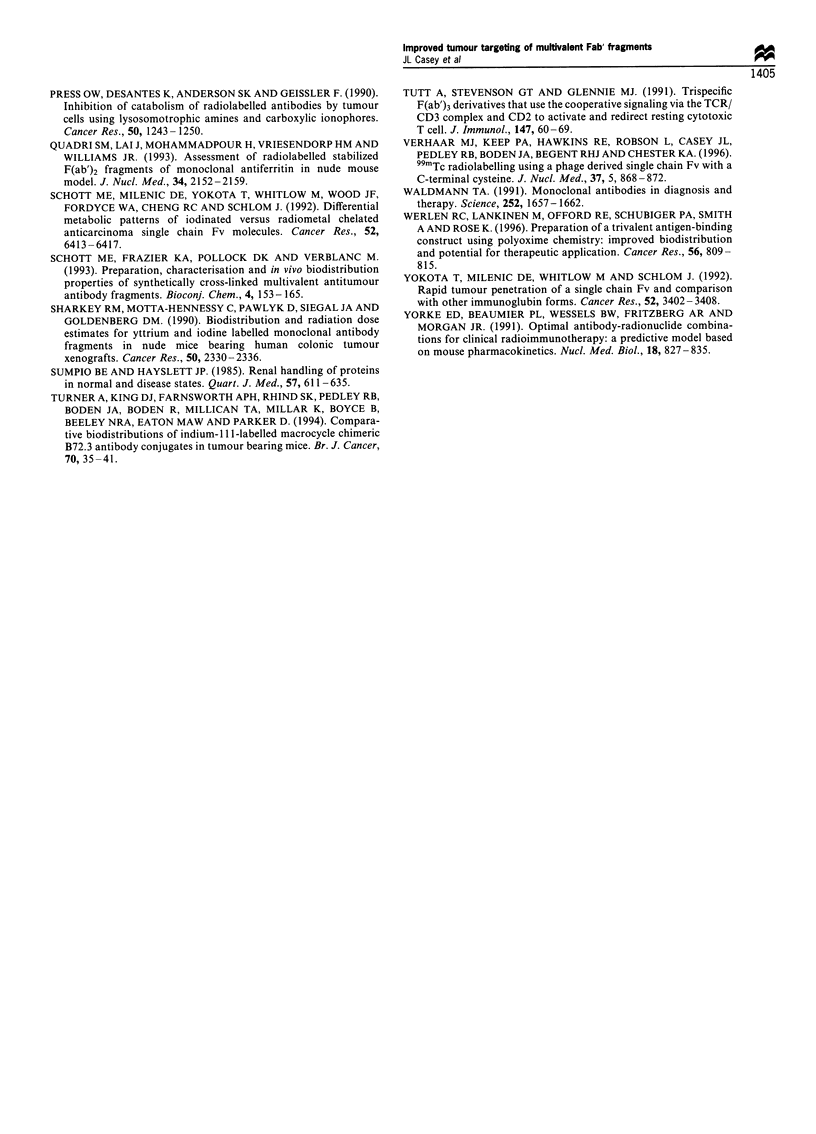# Preparation, characterisation and tumour targeting of cross-linked divalent and trivalent anti-tumour Fab' fragments.

**DOI:** 10.1038/bjc.1996.555

**Published:** 1996-11

**Authors:** J. L. Casey, D. J. King, L. C. Chaplin, A. M. Haines, R. B. Pedley, A. Mountain, G. T. Yarranton, R. H. Begent

**Affiliations:** Department of Clinical Oncology, Royal Free Hospital School of Medicine, London, UK.

## Abstract

**Images:**


British Journal of Cancer (1996) 74, 1397-1405
© 1996 Stockton Press All rights reserved 0007-0920/96 $12.00

### Preparation, characterisation and tumour targeting of cross-linked divalent and trivalent anti-tumour Fab′ fragments

JL Casey[1], DJ King[2], LC Chaplin[2], AMR Haines[2], RB Pedley[1], A Mountain[2], GT Yarranton[2] and RHJ Begent[1]

[1]*Cancer Research Campaign Laboratories, Department of Clinical Oncology, Royal Free Hospital School of Medicine, London NW3 2PF, UK;* [2]*Celltech Therapeutics Ltd, 216 Bath Road, Slough, Berkshire SL1 4EN, UK.*

**Summary** The monoclonal anti-CEA antibody, A5B7, has previously been administered to patients for radioimmunotherapy (RIT). Long circulation time and the formation of an immune response have limited therapeutic success in the clinic. Antibody fragments can be used to reduce the *in vivo* circulation time, but the best combination of fragment and radioisotope to use for therapy is far from clear. In this study we have compared the biodistribution of A5B7 IgG and $F(ab')_2$ with chemically cross-linked divalent (DFM) and trivalent (TFM) A5B7 Fab′ fragments in nude mice bearing human colorectal tumour xenografts. The cross-linkers were designed to allow site-specific labelling using yttrium 90 ($^{90}Y$), a high-energy $\beta$-emitter. We have also compared the above antibody forms conjugated to both $^{131}I$ and $^{90}Y$. Both DFM and TFM were fully immunoreactive and remained intact after radiolabelling and incubation in serum at 37°C for 24 h. Biodistribution results showed similar tumour uptake levels and an identical blood clearance pattern for $F(ab')_2$ and DFM with high tumour–blood ratios generated in each case. However, unacceptably high kidney accumulation for both $F(ab')_2$ and DFM and elevated splenic uptake of DFM labelled with $^{90}Y$ was observed. Kinetic analysis of antigen binding revealed that DFM had the fastest association rate ($k_{ass} = 1.6 \times 10^5$ $Ms^{-1}$) of the antibody forms, perhaps owing to increased flexibility of the cross-linker. This advantage implies that DFM may be more suitable than $F(ab')_2$ radiolabelled with $^{131}I$ for RIT. TFM cleared from the blood significantly faster than A5B7 IgG when labelled with both $^{131}I$ and $^{90}Y$, producing an improved therapeutic tumour–blood ratio. Kidney accumulation was not observed for [$^{90}Y$]TFM, but a slightly higher splenic uptake was observed that may indicate reticuloendothelial system (RES) uptake. Overall, tumour uptake was higher for $^{90}Y$-labelled antibodies than for $^{131}I$-labelled antibodies. Because of the faster clearance, it should be possible to administer a higher total dose of $^{90}Y$-labelled TFM than IgG, which is attractive for RIT. Both A5B7 DFM and TFM, therefore, show favourable properties compared with their parent antibody forms.

**Keywords:** maleimide cross-linking; radioimmunotherapy; Di-Fab; Tri-Fab

The use of antibodies to deliver radiation selectively to tumours for therapeutic and diagnostic purposes is now well established (Waldmann, 1991; Jurcic and Scheinberg., 1994; Larson *et al.*, 1994). The murine A5B7 antibody raised against carcinoembryonic antigen (CEA) has been used for both imaging and therapy in nude mice bearing human colorectal tumour xenografts (Pedley *et al.*, 1993) and in patients (Lane *et al.*, 1994). However, results from clinical trials using this antibody, and many other studies on the treatment of solid tumours, have been disappointing, considering the success in mice (Begent and Pedley, 1990; Delaloye and Delaloye, 1995). In spite of this, some patient responses to radioimmunotherapy (RIT) have been reported (Begent and Pedley, 1990; Lane *et al.*, 1994), suggesting that improvements to current methodology may be clinically beneficial.

There are a number of factors thought to influence antibody localisation to the tumour, which may account for the wide variation in tumour uptake levels between patients (Boxer *et al.*, 1992). These include heterogeneous expression of antigen, variable levels of antibody achieved in the blood, the presence of circulating antigen, tumour vascularisation and the penetration of antibody into tumour tissue. In addition, RIT is often limited by the toxicity of circulating activity, particularly to the bone marrow. In attempts to overcome these problems, several strategies have been attempted to improve the delivery to tumours, while removing circulating radiolabelled antibody more rapidly, in order to reduce the dose to the bone marrow. These include the use of specific clearing regimens either *in vivo* or *ex vivo* (Begent *et al.*, 1987; Norrgren *et al.*, 1993), the use of two-step targeting regimens (Goodwin *et al.*, 1994) and the use of antibody fragments.

$F(ab')_2$ fragments have been widely investigated for RIT, both in animal models and in the clinic (Lane *et al.*, 1994). It has been shown that, in some situations, F(ab′) fragments may be more suitable for RIT than intact IgG labelled with $^{131}I$ (Buchegger *et al.*, 1990; Yorke *et al.*, 1991). However, comparatively few studies have attempted to identify the most suitable fragments for RIT with different isotopes. Small antibody fragments such as Fv and single-chain Fv (scFv) fragments showed improved penetration into tumour tissue (Yokota *et al.*, 1992). ScFv fragments also show favourable tumour–blood ratios and are potentially excellent reagents for tumour imaging (Chester *et al.*, 1994). However, the dose of antibody delivered to the tumour by these rapidly cleared fragments is relatively low and further development is required before a therapeutic tumour dose could be delivered.

We have been evaluating alternative reagents comprising multivalent Fab′s produced by chemical cross-linking. Chemically cross-linked di-Fab (DFM) and tri-Fab (TFM) have been prepared from the mouse–human chimeric Fab′ fragment of B72.3 (King *et al.*, 1994) and an engineered human form of the antibody (King *et al.*, 1995). Biodistribution studies with these antibodies have revealed higher tumour accumulation than seen with scFvs, although clearance from the blood is still rapid. In addition, the choice of antibody fragment for RIT may be different for different isotopes. It is well known that the biodistribution of antibody fragments labelled with radioiodine is very different from those labelled with metallic radionuclides such as $^{111}In$, $^{90}Y$, $^{177}Lu$, $^{67}Cu$ and $^{99m}Tc$ (Brown *et al.*, 1987; Sharkey *et al.*, 1990; Schott *et al.*, 1992). The purpose of this study was, therefore, to determine which was the optimal form of the antibody A5B7 for RIT with the isotopes $^{131}I$ and $^{90}Y$.

Correspondence: JL Casey
Received 25 March 1996; revised 15 May 1996; accepted 29 May 1996

In particular, we have developed methods for the production of murine versions of DFM and TFM of A5B7 to allow biodistribution studies of the murine antibody fragments in the nude mouse xenograft system. Although models of this type have been shown to have many limitations, particularly with regard to relative tumour size and accessibility, they have been shown to generate tumour–normal tissue ratios in mice similar to those observed in humans administered with the same antibody (Begent and Pedley, 1990). The biodistribution of the murine reagents produced here may be more representative of the behaviour of engineered human antibodies in patients than direct mouse studies with the recombinant humanised reagents.

### Material and methods

### *Preparation of F(ab')₂ fragments*

A5B7, (IgG 1) was dialysed into 0.1 M sodium acetate, pH 5.5, containing 3 mM EDTA and concentrated by ultrafiltration using a pressurised stirred cell fitted with a YM10 membrane (Amicon) to 10 mg $ml^{-1}$. Bromelain (5 mg) (Sigma) was activated by incubation with freshly prepared 50 mM cysteine (4 ml) in the same buffer for 30 min at 37°C. Activated bromelain was buffer exchanged on a Sephadex G-25 prepacked PD-10 column (Pharmacia) and the concentration of enzyme determined by absorbance at 280 nm. Digestion was performed by incubation of antibody (approximately 100 mg) with enzyme at a ratio of 50:1 (w/w) with gentle mixing at 37°C. Digestion was periodically monitored by high-performance liquid chromatography (HPLC) gel filtration analysis on a Zorbax GF-250 analytical column (DuPont), at a flow rate of 1 ml $min^{-1}$ with a 0.2 M sodium phosphate, pH 7.0, mobile phase, and was usually complete in 1 h. The reaction mix was adjusted to pH 6.0 with 0.1 M sodium hydroxide and bromelain was rapidly removed at 4°C by loading onto an SP-Sepharose (Pharmacia) column (approximately 30 ml) equilibrated with 0.1 M sodium acetate, pH 6.0. $F(ab')_2$ was eluted with 0.5 M sodium chloride and further purified by gel filtration on a 2 m × 2.6 cm diameter Sephacryl S-200 (Pharmacia) column equilibrated in 0.1 M sodium acetate/0.1 M potassium chloride buffer, pH 6.0, containing 3 mM diethylenetriamine penta-acetic acid (DTPA), at a flow rate of 0.3 ml $min^{-1}$.

### *Cross-linking*

Purified $F(ab')_2$ was concentrated by amicon ultrafiltration to approximately 5 mg $ml^{-1}$ and buffer exchanged to 0.1 M sodium acetate, pH 8.0, containing 2 mM DTPA. $F(ab')_2$ was partially reduced to form Fab' fragments to produce a free hinge thiol for cross-linking, using 2-mercaptoethylamine (Fluka) at a final concentration of 5 mM for 30 min at 37°C. The reducing agent was then removed by desalting on a PD-10 column, and the presence of free thiols was measured by titration with 4',4'-dithiodipyridine (Sigma) and measurement of the thiopyridine released at 324 nm. Cross-linking to DFM was performed by incubating desalted reduced Fab' with 12-N4-macrocycle containing dimaleimide linker CT52, for 2 h at 37°C at a molar ratio of 2:1. TFM was prepared using Fab' in the same way by cross-linking with a 12-N4-macrocycle containing trimaleimide linker CT998, at a 3-fold molar excess of Fab'–linker. Detailed synthesis and chemical structure of the cross-linkers, CT52 and CT998, has been previously described by King *et al.* (1994).

Preparation of antibodies for $^{90}Y$ labelling was performed under metal-free conditions. This was achieved by use of Milli-Q SP deionised water (Millipore), high-quality reagent buffers and metal-free containers. Purification of DFM and TFM after cross-linking was achieved by HPLC gel filtration using a Zorbax GF-250XL column run at a flow rate of 3 ml $min^{-1}$ in 0.2 M sodium phosphate, pH 7.0, containing 2 mM DTPA. To confirm purity, SDS–PAGE under non-reducing conditions was performed. To check for the presence of reoxidised $F(ab')_2$ in the DFM, a small sample of DFM was reduced as above and alkylated with excess *N*-ethlymaleimide. HPLC analysis was used to calculate the percentage of $F(ab')_2$ in the sample relative to DFM.

### *Preparation of IgG and F(ab')₂ macrocycle conjugates*

A5B7 IgG and $F(ab')_2$ were dialysed into 0.1 M sodium phosphate, pH 8.0, containing 2 mM DTPA and incubated with a 10-fold molar excess of 2-iminothiolane hydrochloride (Sigma) for 30 min at room temperature. Unreacted reagent was removed by desalting on a PD-10 column equilibrated with the same buffer at pH 6.0, and a thiol assay was performed. A 3-fold molar excess of CT52 containing the 12-N4-macrocycle group for $^{90}Y$ labelling (Harrison *et al.*, 1991) was added to thiolated antibody or $F(ab')_2$ and incubated for 2 h at room temperature. The number of macrocycles per antibody was determined by measuring the number of thiol groups remaining after addition of macrocycle. Suitable controls revealed that the loss of thiol groups by oxidation over time was negligible.

### *Antigen binding and kinetic analysis*

To determine the immunoreactivity of DFM, TFM and macrocycle conjugates compared with unmodified IgG and $F(ab')_2$ enzyme-linked immunosorbent assays (ELISAs) were performed.

Microtitre plates were coated with 100 $\mu$l of CEA antigen (perchloric acid extracted from a patient and affinity purified in the department of Clinical Oncology) at 2 $\mu g\ ml^{-1}$ in phosphate-buffered saline (PBS, Sigma) for 1 h at room temperature, then blocked with 3% bovine serum albumin (BSA, Sigma) in PBS overnight at 4°C. Serial doubling dilutions (100 $\mu$l) of 10 $\mu g\ ml^{-1}$ DFM, TFM, $F(ab')_2$, IgG and macrocycle conjugates of IgG and $F(ab')_2$ in PBS/0.05% Tween 20, were applied to washed coated plates, for 1 h with gentle mixing. Plates were washed four times with PBS/0.05%Tween 20 and four times with distilled water, and 100 $\mu$l of anti-mouse peroxidase 1:1000 dilution (Amersham) was added and incubated for 1 h with gentle mixing. After washing, 100 $\mu$l of the substrate *O*-phenylenediamine dihydrochloride (OPD, Sigma, 10 mg tablet dissolved in 40 ml of 50 mM citrate buffer, pH 5.0, with 8 $\mu$l of hydrogen peroxide) was applied to each well. After approximately 5 min, the reaction was stopped by the addition of 4 M hydrochloric acid. The optical density at 490 nM was measured using a plate reader (Boots-Celltech Therapeutics). This assay was repeated to ensure consistent results.

Kinetic analysis was carried out by surface plasmon resonance using the BIAcore system (Pharmacia Biosensor) to measure on and off rates of the above antibodies. CEA antigen was immobilised to biosensor chips using surface thiol chemistry or, alternatively, by aldehyde chemistry, and the immobilised antigen density was optimised in a similar way to that described previously (Abraham *et al.*, 1995). Kinetic binding parameters were calculated using BIA evaluation software after correcting concentrations to nM binding sites (assuming two binding sites per antibody for IgG, $F(ab')_2$ and DFM, three for TFM and one binding site for F(ab'), such that antibody forms with different numbers of binding sites could be compared.

### *Radiolabelling and animal studies*

Labelling with $^{131}I$ was performed using the chloramine T method. Free iodine was removed using a PD-10 column blocked with 3% BSA and equilibrated in PBS, and percentage incorporation of radiolabel was analysed by thin-layer chromatography (TLC) analysis in 80% methanol.

Antibodies for $^{90}Y$ labelling were first desalted into 0.1 M MES buffer, pH 6.0, at concentrations >1 mg $ml^{-1}$. $^{90}$Yttrium chloride ($^{90}YCl_3$, Amersham) at 50 mCi $ml^{-1}$ was added to achieve a specific activity of 2 $\mu Ci\ \mu g^{-1}$ and incubated for 20 min at room temperature. The reaction was quenched by addition of 10 mM DTPA for 10 min at room temperature. Incorporation was measured by TLC in a mobile phase of 0.1 M citrate buffer, pH 5.0, and HPLC gel filtration was used to remove any unreacted $^{90}$Y. Characterisation of antibodies after labelling was performed to ensure full immunoreactivity by applying a dilution of the radiolabelled antibody to a 1 ml CEA affinity column and measuring the percentage bound, as described previously by Casey *et al.* (1995). Stability of radiolabelled antibody was analysed by application of an aliquot of the sample to a Sephacryl S-300 column (110 × 1 cm). Fractions (1.3 ml) were monitored for $^{131}$I or $^{90}$Y levels. Stability at 37°C in human serum for 24 h was also assessed.

Comparative biodistribution experiments were performed in the nude mouse colorectal xenograft model, LS174T (Pedley *et al.*, 1993).

### Results

### *Preparation of F(ab′)$_2$, DFM and TFM*

Digestion of A5B7 with bromelain enzyme routinely produced approximately 70% fully immunoreactive F(ab′)$_2$ in 1 h. Purification of this F(ab′)$_2$ resulted in material which was >90% pure as assessed by SDS–PAGE (Figure 1). Cross-linking of Fab′ to DFM was monitored by HPLC gel filtration after a 2 h incubation period and resulted in yields of approximately 40% cross-linked dimer with less than 5% reoxidised F(ab′)$_2$. For TFM cross-linking, yields of approximately 25% cross-linked trimer were obtained. These yields are relatively low compared with those obtained previously with recombinant antibody fragments, where yields of 70% (DFM) and 60% (TFM) have been observed (King *et al.*, 1994). This is probably a result of using murine A5B7 Fab′, which contains three hinge cysteine residues compared with recombinant Fab′, which has a single hinge thiol residue. Optimisation studies have shown that higher cross-linking levels may be achieved on a larger scale at higher antibody concentrations. DFM and TFM were purified by HPLC gel filtration to >90% purity as illustrated by SDS–PAGE analysis in Figure 1.

**Figure 1** Conjugated to macrocycle SDS–PAGE analysis (4–20% gradient) under non-reducing conditions of purified (**a**) DFM, (**b**) TFM, (**c**) F(ab′)$_2$, (**d**) IgG and molecular weight markers. Gel was stained with Coomassie blue.

### *Antigen binding and kinetic analysis*

Antigen binding analysis by ELISA demonstrated full reactivity of DFM and F(ab′)$_2$-macrocycle conjugate compared with that of native F(ab′)$_2$ (Figure 2a). DFM also showed slightly higher binding than F(ab′)$_2$ and the F(ab′)$_2$-macrocycle conjugate. TFM and IgG-macrocycle retained full reactivity (Figure 2b) compared with unmodified IgG, and TFM also demonstrated a slightly higher binding than IgG and the macrocycle conjugate. It was important to analyse the immunoreactivity of antibody after modification, as high levels of reducing agent or 2-iminothiolane have been reported to cause loss of immunoreactivity or aggregation (Turner *et al.*, 1994).

Kinetic analysis of antigen binding was performed using surface plasmon resonance with a BIAcore instrument (Pharmacia Biosensor). The amount of CEA coupled to the sensor chip was optimised in preliminary experiments using either PDEA surface thiol or aldehyde immobilisation. The optimal antigen binding density for kinetic analysis was determined at which the antibody–antigen interaction was minimal for mass transport (768 RU for aldehyde coupling and 2544 RU for surface thiol immobilisation of CEA). Table I shows a comparison of association and dissociation rate constants ($k_{on}$ and $k_{off}$) for the antibodies studied,

**Figure 2** Antigen binding analysis by ELISA of native, conjugated and cross-linked antibodies. (**a**) Native F(ab′)$_2$ (▲), F(ab′)$_2$ conjugated to macrocycle (●), and DFM (■). (**b**) Native IgG (▲), IgG conjugated to macrocycle (●), and TFM (■). The assay was repeated to ensure results were reproducible.

comparing both immobilised CEA surfaces, the values being a mean of four serial dilutations of antibody (333 nM, 167 nM, 83 nM and 42 nM). To compare forms of antibody with different numbers of binding sites, the values have been converted to nM binding sites for each entity. Monovalent Fab′ was included in the evaluation and showed a considerably slower on rate ($k_{ass}$ $3.55 \times 10^4$ mean of values for both surfaces) and faster off rate ($k_{diss}$ $1.25 \times 10^{-4}$ mean) than the multivalent species, presumably owing to the lower avidity of a single binding site compared with the divalent or trivalent antibody forms. The on rates were significantly superior for DFM ($k_{ass}$ $1.6 \times 10^5$ mean) when compared with mean values for both surfaces of F(ab′)$_2$ ($k_{ass}$ $7.34 \times 10^4$), IgG ($k_{ass}$ $5.3 \times 10^4$) and TFM ($k_{ass}$ $7.33 \times 10^4$). However, there was little difference in off rate between the divalent and trivalent antibodies, F(ab′)$_2$ and IgG: ($k_{diss}$ $0.17 \times 10^{-4} - 0.47 \times 10^{-4}$). Surprisingly, the increase in avidity of TFM did not produce higher kinetic binding, although there was a clear advantage between mono- and divalent constructs. The dissociation rate constants measured were close to the lower limit of detection possible with the BIAcore system. In general, the results obtained with the CEA immobilised via surface thiol

**Table I** BIAcore kinetic parameters for antibodies determined by binding to CEA immobilised by either aldehyde coupling or surface thiol chemistry

| | $k_{ass}$ *(1 × E4 Ms$^{-1}$)* | | | $k_{diss}$ *(1 × E-4 s$^{-1}$)* | | |
|---|---|---|---|---|---|---|
| | *Aldehyde* | *Thiol* | *Mean* | *Aldehyde* | *Thiol* | *Mean* |
| Fab′ | 3.25 (±1.3) | 3.84 (±0.6) | 3.55 | 1.44 (±4.0) | 1.06 (±1.4) | 1.25 |
| F(ab′)$_2$ | 5.17 (±1.0) | 9.50 (±1.2) | 7.34 | 0.45 (±0.04) | 0.18 (±0.04) | 0.32 |
| DFM | 12.5 (±2.6) | 19.5 (±2.0) | 16.0 | 0.47 (±2.5) | 0.17 (±0.3) | 0.32 |
| TFM | 5.46 (±1.7) | 9.2 (±2.1) | 7.33 | 0.43 (±2.1) | 0.17 (±1.3) | 0.30 |
| IgG | 3.64 (±1.2) | 6.96 (±1.6) | 5.30 | 0.34 (±0.2) | 0.20 (±0.03 | 0.27 |

Values are corrected for number of binding sites and molecular weight and presented as association and dissociation constants. The values are average (mean) values calculated from analysis of four concentrations of antibody, standard deviations are in brackets. The overall mean values of both couplng methods for $k_{ass}$ and $k_{diss}$ are shown in the 'mean' columns.

**Figure 3** Gel filtration profile of radiolabelled antibodies incubated in human serum for 24 h at 37°C. (**a**) DFM (■) and F(ab′)$_2$ (▲) labelled with $^{131}$I. (**b**) DFM (■) and F(ab′)$_2$ (▲) labelled with $^{90}$Y. (**c**) TFM (■) and IgG (▲) labelled with $^{131}$I. (**d**) TFM (■) and IgG (▲) labelled with $^{90}$Y. Molecular weight markers for 200 kDa and 60 kDa are marked with an arrow. C.p.m is a measure of radioactivity in counts per minute.

chemistry showed faster on rates and slower off rates than for aldehyde immobilisation chemistry shown in Table I. This probably reflects the fact that the antigen density was significantly higher for the thiol-immobilised surface allowing more rebinding events to occur during the dissociation phase than with the alternative surface.

*Radiolabelling and stability*

TLC analysis of radiolabelled antibodies routinely revealed 96–99% incorporation of $^{131}$I and $^{90}$Y after purification. Antigen binding by application of a small sample of radiolabelled antibody to a 1 ml CEA affinity column revealed retention of 90–96% binding. A non-CEA antibody was radiolabelled as a control and 2–10% total activity bound to the column.

Antibodies after labelling and incubation at 37°C in normal human serum remained intact and non-aggregated, as shown in Figure 3 by S-300 gel filtration chromatography.

*Biodistribution study*

The biodistribution of A5B7 F(ab′)$_2$ and DFM, and also A5B7 IgG and TFM were compared in tumour-bearing mice over a 6 day period after radiolabelling with both $^{131}$I and $^{90}$Y.

*F(ab′)$_2$ and DFM*

The tissue distributions of $^{131}$I- and $^{90}$Y-labelled F(ab′)$_2$ and DFM were compared at 3, 24, 48 and 144 h time points. The biodistribution of A5B7 F(ab′)$_2$ and DFM labelled with $^{131}$I (Figure 4a and b) proved to be very similar, suggesting there is no significant difference in the stability of these fragments *in vivo*. Rapid clearance from the blood and normal tissues by 24 h produced high therapeutic tumour–blood ratios illustrated in Table II (38:1 F(ab′)$_2$; 26:1 DFM), which were

**Table II** Tumour to blood ratios of percentage injected dose at various time intervals after injection of radiolabelled antibody

| | *3 h* | *24 h* | *48 h* | *144 h* |
|---|---|---|---|---|
| [$^{131}$I]F(ab′)$_2$ | 0.8 | 38 | 50 | 71 |
| [$^{131}$I]DFM | 1.0 | 26 | 43 | 61 |
| [$^{90}$Y]F(ab′)$_2$ | 0.9 | 23 | 22 | 5.0 |
| [$^{90}$Y]DFM | 0.9 | 40 | 27 | 7.0 |
| [$^{131}$I]IgG | 0.3 | 2.0 | 4.3 | 23 |
| [$^{131}$I]TFM | 0.5 | 6.0 | 12 | 44 |
| [$^{90}$Y]IgG | 0.4 | 4.0 | 5.8 | 17 |
| [$^{90}$Y]TFM | 0.4 | 15 | 15 | 14 |

Data are expressed as a mean of four mice.

**Figure 4** Biodistribution of (**a**) [$^{131}$I]F(ab′)$_2$, (**b**) [$^{131}$I]DFM, (**c**) [$^{90}$Y]F(ab′)$_2$ and (**d**) [$^{90}$Y]DFM in nude mice bearing LS174T human tumour xenografts. Time points at 3 h (first column), 24 h (second column), 48 h (third column) and 144 h (fourth column) after i.v. injection. Results are expressed as percentage injected dose per gram of tissue; columns are a mean of four mice and bars represent standard deviations.

**Figure 5** Biodistribution of (**a**) $^{131}$I[IgG], (**b**) $^{131}$I[TFM], (**c**) $^{90}$Y[IgG] and (**d**) $^{90}$Y[TFM] in nude mice bearing LS174T human tumour xenografts. Time points at 3 h (first column), 24 h (second column), 48 h (third column) and 144 h (fourth column) after i.v. injection. Results are expressed as percentage of injected dose per gram of tissue; columns are a mean of four mice and bars represent standard deviations.

retained over the 6 day period. Labelling with $^{90}$Y dramatically altered the biodistribution (Figure 4c and d) leading to very high accumulation in the kidney at early time points and throughout the 6 days, producing a very different clearance pattern to the iodinated fragments. High splenic uptake was also observed, which accumulated over time, especially for DFM (144 h: F(ab′)$_2$ 8.5%; DFM 20%). Despite unfavourable uptake in the kidney and spleen, tumour uptake levels were similar to the iodinated fragments (48 h: [$^{90}$Y]DFM 10%, [$^{131}$I]DFM 6% injected dose per gram).

*IgG and TFM*

The blood clearance of TFM, despite its similar size (150 kDa), was significantly faster than IgG ($P<0.02$, using the Mann–Whitney *U*-test non-parametric statistical analysis) as illustrated in Figure 5. This produced superior therapeutic tumour–blood ratios (Table II) at 24 h $^{131}$I (IgG 2:1; TFM 6:1), $^{90}$Y (IgG 4:1; TFM 15:1) and 48 h $^{131}$I (IgG 4:1; TFM 12:1), $^{90}$Y (IgG 5.8:1; TFM 15:1). The levels of activity accumulated at the tumour were increased by labelling with $^{90}$Y (Figure 5c and d); this persisted over time and was most apparent at later time points, e.g. at 48 h: $^{131}$I (IgG 15% ID $g^{-1}$; TFM 10% ID $g^{-1}$), $^{90}$Y (IgG 25% ID $g^{-1}$; TFM 17% ID $g^{-1}$). This may be caused by higher retention of yttrium-labelled conjugates in cells compared with iodinated conjugates that are dehalogenated. The levels of radiolabel in the tumour for IgG and TFM were also greater than for F(ab′)$_2$ and DFM fragments, probably as a result of slower clearance from the circulation. However, the lower molecular weight fragments produced high therapeutic ratios earlier, potentially reducing toxicity and, thus, allowing larger doses to be given. In contrast to $^{90}$Y-labelled DFM and F(ab′)$_2$, kidney uptake levels of both [$^{90}$Y]IgG and [$^{90}$Y]TFM were relatively low. However, referring to the 144 h time point, it does appear that a greater proportion of [$^{90}$Y]TFM clears through the kidney than [$^{90}$Y]IgG (TFM 9%, IgG 3.4%), and this is also reflected by the higher values in the liver for [$^{90}$Y]IgG at this time point (TFM 4.4%, IgG 12.8%). Slightly higher splenic and femur uptake was observed for the [$^{90}$Y]TFM compared with [$^{90}$Y]IgG, which may indicate non-specific uptake by the reticuloendothelial system (RES).

### Discussion

It has been widely documented that antibody fragments injected into tumour-bearing nude mice give higher tumour to normal tissue ratios than the parent IgG (Buchegger *et al.*, 1988; Pedley *et al.*, 1993). Removal of the Fc portion may also be beneficial owing to the removal of binding sites for Fc receptors, thus lowering toxicity (Buchegger *et al.*, 1992).

However, despite these potential benefits of using $F(ab')_2$ fragments, the lack of the Fc portion exposes the hinge regoin, making it more susceptible to enzymatic and/or reductive breakdown. In addition, different $F(ab')_2$ fragments have different *in vivo* stabilities. For example, in a study comparing murine $F(ab')_2$ from an $IgG_1$ antibody with chimeric $F(ab')_2$ of human subclasses 1, 2 and 4, it was reported that human $IgG_4$ $F(ab')_2$ fragments were relatively unstable *in vivo*, human $IgG_2$ the most stable and murine $IgG_1$ and human $IgG_1$ $F(ab')_2$ of intermediate stability (Buchegger *et al.*, 1992). Chemically cross-linked $F(ab')_2$ fragments and trispecific $F(ab')_3$ derivatives have been produced through use of bis-maleimide linkers using techniques developed by Glennie *et al.* (1987) and Tutt *et al.* (1991). Recombinant Fab′s have also been synthesised with a single hinge thiol to facilitate cross-linking reactions (Carter *et al.*, 1992; King *et al.*, 1994) and scFv molecules with a C-terminal cysteine expressed, which allow cross-linking (Cumber *et al.*, 1992). In some reports, increased *in vivo* stability of the cross-linked fragments has been demonstrated (Quadri *et al.*, 1993; King *et al.*, 1994). However, as $F(ab')_2$ fragments vary in their *in vivo* stability themselves, the nature of the $F(ab')_2$ fragments must be taken into account and direct comparisons in the same system are necessary. In this study, we have shown that A5B7 chemically cross-linked DFM and native $F(ab')_2$ fragments have similar *in vitro* and *in vivo* stability. Although higher splenic uptake was observed for $[^{90}Y]$DFM compared with $[^{90}Y]F(ab')_2$, there was no significant difference in levels of activity in tissues, including blood, kidney and tumour when labelled with either $^{131}I$ or $^{90}Y$.

The major site of antibody fragment catabolism in mice is the kidneys (Covell *et al.*, 1986) and this was the normal organ with the highest level of $^{131}I$ and $[^{90}Y]F(ab')_2$ and DFM at early time points. Radioiodinated fragments undergo metabolism to release low molecular weight fragments, which are rapidly released from cells and cleared (Press *et al.*, 1990), whereas radiometals are retained longer owing to cellular trapping of the chelate (Pimm *et al.*, 1989). Biodistribution of antibody fragments labelled with $^{131}I$ and $^{111}In$ have been compared previously (Andrew *et al.*, 1988). For $^{111}In$-labelled $F(ab')_2$ >60% of administered activity was retained in the kidneys compared with <10% for $[^{131}I]F(ab')_2$. This is similar to the difference we observe here for $^{90}Y$-labelled $F(ab')_2$ and DFM; kidney–blood ratios are increased 3-fold at 3 h and over 100-fold at 24 h compared with $^{131}I$-labelled fragments.

The stability of the attachment of radiochelate to antibody is an important determinant of therapeutic ratio, since some labelling methods, involving derivatives of the chelating ligand DTPA, produce relatively labile conjugates *in vivo* (Harrison *et al.*, 1991). Weak ligands will lead to both a reduction in the amount of $^{90}Y$-conjugate in the tumour and irradiation of the normal tissues with free $^{90}Y$, especially proliferating tissues of the bone marrow, since $^{90}Y$ is a bone-seeking isotope (Hnatowich *et al.*, 1983). The development of new chelating agents, such as the macrocyclic chelating agent DOTA (Moi *et al.*, 1988; Cox *et al.*, 1989), have proved to be more stable *in vivo* (Hird *et al.*, 1991; Harrison *et al.*, 1991). The cross-linkers used in this study contain the 12N4 DOTA macrocycle, which allows the site-specific attachment of $^{90}Y$ and this has the added advantage that there is no loss of antigen binding after radiolabelling. Attachment of radiolabel in random positions, as we describe here for IgG and $F(ab')_2$, leads to non-homogenous preparations owing to variation in the number of thiol groups generated, and may reduce immunoreactivity, if residues which contribute to antigen binding are modified.

Engineering an additional C-terminal cysteine residue on scFv or Fab′ subunits and thus providing a free thiol group, may also be useful for site-specific radiolabelling with technetium-99m (Verhaar *et al.*, 1996), again allowing retention of immunoreactivity as a result of conjugation distant from the antigen binding sites.

The increase in valency and, therefore, avidity of divalent fragments over monovalent fragments produced a significant increase in kinetic association and dissociation rate-binding parameters. However, the increase in valency from divalent to trivalent molecules did not further increase kinetic binding. Three Fab′ arms may not all be accessible for binding at the same time to a solid surface. Therefore, steric hindrance to antigen sites on solid surface may explain why an increase in association and dissociation rate was not achieved. A cell binding assay may be more representative of binding parameters *in vivo*; in a previous study improved binding of trivalent molecules over divalent $F(ab')_2$ was demonstrated using this type of assay (Werlen *et al.*, 1996).

The biodistribution of IgG and TFM in the xenograft model showed surprisingly faster clearance for TFM compared with whole antibody, despite having the same molecular weight. The same phenomenom has been observed with trivalent bis-maleimide-linked Fab′ fragments described previously (King *et al.*, 1994; Schott *et al.*, 1993), and may be partially caused by the lack of the Fc portion thought to be responsible for the long circulation time of the intact immunoglobulin. Tumour accumulation of TFM was greater than for the divalent fragments, and similar levels to IgG were observed up to 24 h. The major advantage of faster clearance leading to lower blood toxicity is that for therapy a larger dose may be given. Again, by labelling with $^{90}Y$, tumour retention is superior than for iodinated IgG and TFM owing to the absence of dehalogenation and/or $^{131}I$ metabolism. Several publications (Schott *et al.*, 1992; Sharkey *et al.*, 1990) have also reported this finding, which could also explain the higher percentage injected dose of the $^{90}Y$-labelled antibodies in normal tissues, such as kidney spleen and liver. The splenic uptake of $^{90}Y$-labelled DFM and TFM was notably higher than the $F(ab')_2$ and IgG. This indicates that there may be some RES uptake of the cross-linked fragments. This will require further investigation and dosimetry evaluation with higher doses of radioconjugate.

In contrast to the divalent fragments, kidney accumulation was greatly reduced. This is presumably mainly due to the increase in molecular weight, although the slight increase in kidney levels of TFM compared with IgG suggest that there may be other factors, such as shape and charge, which also influence the filtration process (Sumpio and Hayslett, 1985). Catabolism of TFM to form $F(ab')_2$ or Fab′ fragments could also occur, which may also contribute to increased kidney uptake. However, there is no evidence to suggest reabsorption of fragmented TFM or DFM, or breakdown occurring in the serum, by HPLC analysis of serum from mice injected with labelled DFM and TFM (unpublished finding).

In the present study, DFM showed faster blood clearance than TFM and IgG and similar biodistribution to $F(ab')_2$ in the xenograft model. High kidney accumulation of both $[^{90}Y]$DFM and $[^{90}Y]F(ab')_2$ and increased splenic uptake of $[^{90}Y]$DFM is clearly unacceptable for RIT. However, this high kidney and splenic uptake was not seen on labelling with $^{131}I$. DFM has a faster $k_{on}$ rate than $F(ab')_2$ and all the other antibody forms, which may be a consequence of the increased spacing or flexibility of the chemical cross-linker. Therefore, although the biodistribution data demonstrate equivalent tumour uptake levels, we conclude that A5B7 DFM, owing to its faster $k_{on}$ rate, may be more suitable than $F(ab')_2$ when radiolabelled with $^{131}I$ for RIT.

TFM clears faster from the blood than IgG and produces higher tumour uptake and lower kidney accumulation than DFM and $F(ab')_2$ radiolabelled with $^{90}Y$. Although there is a slight elevation in splenic uptake, this combination avoids long blood circulation time, which is dose limiting, and spares the kidneys from damage by radiation accumulation. Therefore, from these results we conclude that $^{90}Y$-labelled A5B7 TFM would be the most suitable antibody species for RIT.

The superior pharmacokinetics of murine versions of DFM and TFM compared with the parent IgG and $F(ab')_2$ *in vivo*, coupled with the ability to re-treat patients by using humanised forms, should provide improved clinical therapy in the future. A humanised Fab' version of A5B7 has been constructed (Adair *et al.*, 1992) and preliminary data involving hDFM and hTFM are promising.

**Acknowledgements**
The authors would like to thank JA Boden and R Boden for technical assistance. This work was funded by an MRC LINK grant in association with Celltech Therapeutics Ltd.

### References

ABRAHAM R, BUXBAUM S, LINK J, SMITH R, VENTI C AND DARSLEY M. (1995). Screening and kinetic analysis of recombinant anti-CEA antibody fragments. *J. Immunol. Methods*, **183**, 119–125.

ADAIR JA, BODMER MW, MOUNTAIN A AND OWENS RJ (1992). CDR grafted anti-CEA antibodies and their production. *World patient application.* WO 92/01059.

ANDREW SA, PERKINS AC, PIMM MV AND BALDWIN W. (1988). A comparison of iodine and indium labelled anti CEA intact antibody, $F(ab')_2$ and Fab' fragments by imaging tumour xenografts. *Eur. J. Nucl. Med.*, **13**, 598–604.

BEGENT RHJ AND PEDLEY RB. (1990). Antibody targeted therapy in cancer: comparison of murine and clinical studies. *Cancer Treat. Rev.*, **17**, 373–378.

BEGENT RHJ, BAGSHAWE KD, PEDLEY RB, SEARLE F, LEDERMANN JA, GREEN AJ, KEEP PA, CHESTER KA, GLASER MG AND DALE RG. (1987). Use of second antibody in radioimmunotherapy. *NCI Monogr.*, **31**, 1035–1044.

BOXER GM, BEGENT RHJ, KELLY AMB, SOUTHALL PJ, BLAIR SB, THEODOROU NA, DAWSON PM AND LEDERMANN JA. (1992). Factors influencing variability of localisation of antibodies to carcinoembryonic antigen (CEA) in patients with colorectal cancer–implications for radioimmunotherapy. *Br. J. Cancer*, **65**, 825–831.

BROWN BA, COMEAU RD, JONES PL, LIBERTORE FA, NEACY WP, SANDS H AND GALLAGER BM. (1987). Pharmacokinetics of the monoclonal antibody B72.3 and its fragments labelled with either $^{125}I$ or $^{111}In$. *Cancer Res.*, **47**, 1149–1154.

BUCHEGGER F, VACCA A, CARREL S, SCHREYER M AND MACH JP. (1988). Radioimmunotherapy of human colon carcinoma by $^{131}I$-labelled monoclonal anti-CEA antibodies in a nude mouse model. *Int. J. Cancer*, **41**, 127–134.

BUCHEGGER F, PELEGRIN A, DELALOYE B, BISCHOFF-DELALOYE A AND MACH JP. (1990). Iodine-131 labelled Mab $F(ab')_2$ fragments are more effective and less toxic than intact anti-CEA antibodies in radioimmunotherapy of large human colon carcinoma grafted in nude mice. *J. Nucl. Med.*, **31**, 1035–1044.

BUCHEGGER F, PELEGRIN A, HARDMAN N, HEUSSER C, DOLCI W AND MACH JP. (1992). Different behaviour of mouse–human chimeric antibody $F(ab')_2$ fragments of $IgG_1$, $IgG_2$ and $IgG_4$ subclass *in vivo. Int. J. Cancer*, **50**, 416–422.

CARTER P, KELLEY RF, RODRIQUES ML, SNEDCOR B, COVARRUBIAS M, VELLIGAN MD, WONG WLT, ROWLAND AM, KOTTS CE, CARVER ME, YANG M, BOURELL JH, SHEPARD HM AND HENNER D. (1992). High level *Escherichia coli* expression and production of a bivalent humanised antibody fragment. *BioTechnology*, **10**, 163–167.

CASEY JL, CHESTER KA, ROBSON L, HAWKINS, RE AND BEGENT RHJ. (1995). Purification of bacterially expressed single chain antibodies for clinical applications using metal chelate chromatography. *J. Immunol. Methods*, **179**, 105–116.

CHESTER KA, BEGENT RHJ, ROBSON L, KEEP PA, PEDLEY RB, BODEN JA, BOXER GM, GREEN AJ, WINTER G, COCHET O AND HAWKINS RE. (1994). Phage libraries for the generation of clinically useful antibodies. *Lancet*, **343**, 455–457.

COVELL DG, BARBET J, HOLTON OD, BLACK CDV, PARKER RJ AND WEINSTEIN JN. (1986). Pharmacokinetics of monoclonal immunoglobulin $G_1$, $F(ab')_2$, and Fab' in mice. *Cancer Res.*, **46**, 3969–3978.

COX JP, JANKOWSKI KJ, KATAKY R, PARKER D, BEELEY NRA, BOYCE BA, EATON MAW, MILLAR K, MILLICAN AT, HARRISON A AND WALKER C. (1989). Synthesis of a kinetically stable yttrium-90 labelled macrocycle-antibody conjugate. *J. Chem. Soc. Chem. Commun.*, **1989**, 797–798.

CUMBER AJ, WARD ES, WINTER G, PERNELL GD AND WARWRZNCZAK EJ. (1992). Comparative stabilities *in vitro* and *in vivo* of a recombinant mouse antibody FvCys fragment and a bisFvCys conjugate. *J. Immunol.*, **149**, 120–126.

DELALOYE AB AND DELALOYE B. (1995). Radiolabelled monoclonal antibodies in tumour imaging and therapy: out of fashion? *Eur. J. Nucl. Med.*, **22**, 571–580.

GLENNIE MJ, MCBRIDE HM, WORTH AT AND STEVENSON GT. (1987). Preparation and performance of bispecific $F(ab'\gamma)_2$ antibody containing thioether-linked Fab'γ fragments. *J. Immunol.*, **7**, 2367–2375.

GOODWIN DA, MEARES CF, WATANABE N, MCTIGUE M, CHAOVAPONG W, RANSONE CM, RENN O, GREINER DP, KUKIS DL AND KRONENBERGER SI. (1994). Pharmacokinetics of a pretargeted monoclonal antibody 2D12.5 and $^{88}Y$-Janus-2-(p-nitrobenzyl)-1,4,7,10-tetraazacyclododecanetetraacetic acid (DOTA) in BALB/c mice with KHJJ adenocarcinoma: a model for radioimmunotherapy. *Cancer Res.*, **54**, 5937–5946.

HARRISON A, WALKER CA, PARKER D, JANKOWSKI KJ, COX JPL, CRAIG AS, SANSOM JM, BEELEY NRA, BOYCE RA, CHAPLIN L, EATON AW, FARNSWORTH APH, MILLAR K, MILLICAN AT, RANDALL AM, RHIND SK, SECHER DS AND TURNER A. (1991). The *in vivo* release of $^{90}Y$ from cyclic and acyclic ligand-antibody conjugates. *Nucl. Med. Biol.*, **18**, 469–476.

HIRD V, VERHOEYEN M, BADLEY RA, PRICE D, SNOOK D, KOSMAS C, GOODEN C, BAMIAS A, MEARES CF, LAVENDER JP AND EPENETOS AA. (1991). Tumour localisation with a radioactivity labelled reshaped human monoclonal antibody. *Br. J. Cancer*, **64**, 911–914.

HNATOWICH DJ, CHILDS RL, LANTEIGNE D AND NAJAFI A. (1983). The preparation of DTPA-coupled antibodies radiolabelled with metallic radionuclides: an improved method. *J. Immunol. Methods*, **65**, 147–152.

JURCIC JG AND SCHEINBERG DA. (1994). Recent developments in the radioimmunotherapy of cancer. *Curr. Opin. Immunol.*, **6**, 715–721.

KING DJ, TURNER A, FARNSWORTH APH, ADAIR JR, OWENS RJ, PEDLEY RB, BALDOCK D, PROUDFOOT KA, LAWSON ADG, BEELEY NRA, MILLAR K, MILLICAN A, BOYCE BA, ANTONIW P, MOUNTAIN A, BEGENT RHJ, SHOCAT D AND YARRANTON GT. (1994). Improved tumour targeting with chemically cross-linked recombinant antibody fragments. *Cancer Res.*, **54**, 6176–6185.

KING DJ, ANTONIW P, OWENS RJ, ADAIR JR, HAINES AMR, FARNSWORTH APH, FINNEY H, LAWSON ADG, LYONS A, BAKER TS, BALDOCK D, MACKINTOSH J, GOFTON C, YARRANTON GT, MCWILLIAMS W, SHOCAT D, LEICHNER PK, WELT S, OLD LJ AND MOUNTAIN A. (1995). Preparation and preclinical evaluation of humanised A33 immunoconjugates for radioimmunotherapy. *Br. J. Cancer*, **72**, 1364–1372.

LANE DM, EAGLE KF, BEGENT RHJ, HOPE-STONE LD, GREEN AJ, CASEY JL, KEEP PA, KELLY AMB, LEDERMANN JA, GLASER MG AND HILSON AJW. (1994). Radioimmunotherapy of metastatic colorectal tumours with iodine-131-labelled antibody to carcinoembryonic antigen: phase I/II study with comparative biodistribution of intact and $F(ab')_2$ antibodies. *Br. J. Cancer*, **70**, 521–525.

LARSON SM, DIVGI CR AND SCOTT AM. (1994). Overview of clinical radioimmunodetection of human tumours. *Cancer*, **73**, 832–835.

MOI MK, MEARES CF AND DENARDO SJ. (1988). The peptide way to macrocycle bifunctional chelating agents: synthesis of 2-(p-nitrobenzyl)-1,4,7,10-tetraazacyclododecane-*N,N′,N″, N‴*-tetra-acetid acid, and study of its yttrium (III) complex. *J. Am. Chem. Soc.*, **110**, 6266–6267.

NORRGREN K, STRAND SE, NILSSON R, LINDGREN L AND SJOGREN HO. (1993). A general, extracorporeal method to increase the tumour to normal tissue ratio in radioimmunoimaging and radioimmunotherapy. *J. Nucl. Med.*, **34**, 448–454.

PEDLEY RB, BODEN JA, BODEN R, DALE R AND BEGENT RHJ. (1993). Comparative radioimmunotherapy using intact or $F(ab')_2$ fragments of $^{131}I$ anti-CEA antibody in a colonic xenograft model. *Br. J. Cancer*, **68**, 69–73.

PIMM MV, ANDREW SM AND BALDWIN RW. (1989). Blood and tissue kinetics of radiolabelled anti-CEA monoclonal antibody and $F(ab')_2$ and Fab fragments in nude mice with human tumour xenografts: implications for tumour imaging and radioimmunotherapy. *Nucl. Med. Commun.*, **10**, 585–593.

PRESS OW, DESANTES K, ANDERSON SK AND GEISSLER F. (1990). Inhibition of catabolism of radiolabelled antibodies by tumour cells using lysosomotrophic amines and carboxylic ionophores. *Cancer Res.*, **50**, 1243–1250.

QUADRI SM, LAI J, MOHAMMADPOUR H, VRIESENDORP HM AND WILLIAMS JR. (1993). Assessment of radiolabelled stabilized $F(ab')_2$ fragments of monoclonal antiferritin in nude mouse model. *J. Nucl. Med.*, **34**, 2152–2159.

SCHOTT ME, MILENIC DE, YOKOTA T, WHITLOW M, WOOD JF, FORDYCE WA, CHENG RC AND SCHLOM J. (1992). Differential metabolic patterns of iodinated versus radiometal chelated anticarcinoma single chain Fv molecules. *Cancer Res.*, **52**, 6413–6417.

SCHOTT ME, FRAZIER KA, POLLOCK DK AND VERBLANC M. (1993). Preparation, characterisation and *in vivo* biodistribution properties of synthetically cross-linked multivalent antitumour antibody fragments. *Bioconj. Chem.*, **4**, 153–165.

SHARKEY RM, MOTTA-HENNESSY C, PAWLYK D, SIEGAL JA AND GOLDENBERG DM. (1990). Biodistribution and radiation dose estimates for yttrium and iodine labelled monoclonal antibody fragments in nude mice bearing human colonic tumour xenografts. *Cancer Res.*, **50**, 2330–2336.

SUMPIO BE AND HAYSLETT JP. (1985). Renal handling of proteins in normal and disease states. *Quart. J. Med.*, **57**, 611–635.

TURNER A, KING DJ, FARNSWORTH APH, RHIND SK, PEDLEY RB, BODEN JA, BODEN R, MILLICAN TA, MILLAR K, BOYCE B, BEELEY NRA, EATON MAW AND PARKER D. (1994). Comparative biodistributions of indium-111-labelled macrocycle chimeric B72.3 antibody conjugates in tumour bearing mice. *Br. J. Cancer*, **70**, 35–41.

TUTT A, STEVENSON GT AND GLENNIE MJ. (1991). Trispecific $F(ab')_3$ derivatives that use the cooperative signaling via the TCR/CD3 complex and CD2 to activate and redirect resting cytotoxic T cell. *J. Immunol.*, **147**, 60–69.

VERHAAR MJ, KEEP PA, HAWKINS RE, ROBSON L, CASEY JL, PEDLEY RB, BODEN JA, BEGENT RHJ AND CHESTER KA. (1996). $^{99m}Tc$ radiolabelling using a phage derived single chain Fv with a C-terminal cysteine. *J. Nucl. Med.*, **37**, 5, 868–872.

WALDMANN TA. (1991). Monoclonal antibodies in diagnosis and therapy. *Science*, **252**, 1657–1662.

WERLEN RC, LANKINEN M, OFFORD RE, SCHUBIGER PA, SMITH A AND ROSE K. (1996). Preparation of a trivalent antigen-binding construct using polyoxime chemistry: improved biodistribution and potential for therapeutic application. *Cancer Res.*, **56**, 809–815.

YOKOTA T, MILENIC DE, WHITLOW M AND SCHLOM J. (1992). Rapid tumour penetration of a single chain Fv and comparison with other immunoglubin forms. *Cancer Res.*, **52**, 3402–3408.

YORKE ED, BEAUMIER PL, WESSELS BW, FRITZBERG AR AND MORGAN JR. (1991). Optimal antibody-radionuclide combinations for clinical radioimmunotherapy: a predictive model based on mouse pharmacokinetics. *Nucl. Med. Biol.*, **18**, 827–835.